# A multistage multimodal deep learning model for disease severity assessment and early warnings of high-risk patients of COVID-19

**DOI:** 10.3389/fpubh.2022.982289

**Published:** 2022-11-22

**Authors:** Zhuo Li, Ruiqing Xu, Yifei Shen, Jiannong Cao, Ben Wang, Ying Zhang, Shikang Li

**Affiliations:** ^1^School of Computer Science and Technology, Chongqing University of Posts and Telecommunications, Chongqing, China; ^2^Department of Computing, The Hong Kong Polytechnic University, Kowloon, Hong Kong SAR, China; ^3^Department of Computer Science, The University of Sheffield, Sheffield, United Kingdom; ^4^Baoding No. 2 Central Hospital, Baoding, China; ^5^Chongqing Public Health Medical Center, Chongqing, China

**Keywords:** COVID-19, disease severity assessment, disease progression prediction, sequential stage-wise learning, multimodal feature fusion

## Abstract

The outbreak of coronavirus disease 2019 (COVID-19) has caused massive infections and large death tolls worldwide. Despite many studies on the clinical characteristics and the treatment plans of COVID-19, they rarely conduct in-depth prognostic research on leveraging consecutive rounds of multimodal clinical examination and laboratory test data to facilitate clinical decision-making for the treatment of COVID-19. To address this issue, we propose a multistage multimodal deep learning (MMDL) model to (1) first assess the patient's current condition (i.e., the mild and severe symptoms), then (2) give early warnings to patients with mild symptoms who are at high risk to develop severe illness. In MMDL, we build a sequential stage-wise learning architecture whose design philosophy embodies the model's predicted outcome and does not only depend on the current situation but also the history. Concretely, we meticulously combine the latest round of multimodal clinical data and the decayed past information to make assessments and predictions. In each round (stage), we design a two-layer multimodal feature extractor to extract the latent feature representation across different modalities of clinical data, including patient demographics, clinical manifestation, and 11 modalities of laboratory test results. We conduct experiments on a clinical dataset consisting of 216 COVID-19 patients that have passed the ethical review of the medical ethics committee. Experimental results validate our assumption that sequential stage-wise learning outperforms single-stage learning, but history long ago has little influence on the learning outcome. Also, comparison tests show the advantage of multimodal learning. MMDL with multimodal inputs can beat any reduced model with single-modal inputs only. In addition, we have deployed the prototype of MMDL in a hospital for clinical comparison tests and to assist doctors in clinical diagnosis.

## 1. Introduction

Since December 2019, a novel viral pneumonia caused by severe acute respiratory syndrome coronavirus 2 (SARS-CoV-2), also known as coronavirus disease 2019 (COVID-19) (1–3), first occurred in Wuhan, Hubei Province, China (4), then swept the globe very quickly. As of Sep 1, 2022, data from the World Health Organization (WHO) revealed more than 600 million infections confirmed worldwide with approximately 6.45 million deaths since the outbreak of COVID-19 (5). In view of its strong infectivity and high mortality, WHO declared the pandemic as a Public Health Emergency of International Concern (6).

In practice, the clinical manifestations of COVID-19 vary diversely from asymptomatic, mild infection to severe symptoms (4, 7–9). According to clinical statistics, the majority of COVID-19 cases are mild, and only approximately 5% of the total patients (a part of severe cases) require admission to ICU (10, 11). One of the serious problems we are facing is that the surge of COVID-19 infections leads to rapid depletion of the limited medical resources. The fact is that most of the mild patients can heal without supportive treatment (2, 3), and only a small proportion of them will progress toward severe illness. However, patients whose condition subsequently deteriorate are more prone to be older adults with comorbidities of diabetes, hypertension, cardiac disease, obesity etc. (9, 12), Once the illness changes for the worse, the mortality rate increases significantly, moreover, treating critical patients consumes more medical resources and takes longer treatment courses.

During COVID-19 treatment, doctors perform clinical examinations and laboratory tests on patients every few days. Hence, in every round of the tests, massive multimodal (i.e., various types or categories) clinical data are generated, including the patient demographics, clinical manifestation, laboratory outcomes, the use of drugs and medication, etc. Naturally, it is of great importance that we quickly and accurately distinguish mild and severe patients on admission, then identify those mild cases who are at high risk of turning for the worse in the future based on clinical data analysis and modeling. As a result, early intervention can be taken to prevent mild patients from deterioration.

In the past decade, AI and big data technologies have been widely applied in healthcare and medication and made remarkable achievements (13), which also play an important role in COVID-19 prevention and containment, including screening, testing, contact tracing, treatment and vaccination, and drug development (14–16). So far various forecasting models have been developed for the diagnosis and prognosis of COVID-19 (17–19), which leveraged X-ray and CT images (20–22), clinical characteristics (23, 24), blood test results (25), etc., for model development.

Most of the existing literature for the diagnosis and prognosis of COVID-19 simply makes use of one or two modalities of clinical data, which fails to explore the complementary information provided by multimodal sources. Moreover, the prognostic model is purely based on a single round of lab test results and cannot track the disease progression since onset. To address the characteristics of the consecutive rounds of multimodal clinical test data, in this paper, we propose a multistage multimodal deep learning (MMDL) model to (1) first assess the disease severity, and (2) identify those who are at an early stage of illness and are likely to grow worse. In MMDL, we conceive and implement a sequential stage-wise learning architecture, which abandons the classic structure of RNN (26, 27)/LSTM (28). It is because most patients take no more than five rounds of exams and lab tests before they recover from COVID-19 and are discharged from the hospital, so if we insist on using the RNN/LSTM (Recurrent Neural Network/Long Short-Term Memory) model, the input time step of RNN/LSTM is too few to forecast the future. The design philosophy of MMDL is motivated by the sequence-to-sequence (seq2seq) model (29, 30) in contextual sequence prediction, which extracts the latent feature of one sequence (encoder) and turns it to another sequence (decoder), then the decoded word in a sentence is based on the output from its previous contexts. Concretely, the embodiment of sequential stage-wise learning incorporates the input of the latest round of multimodal clinical data and the past information, and higher weights are given to recent inputs because it has direct influences on the final assessment and prediction results. In each round, to extract the feature of the multimodal clinical data, we design a two-layer multimodal feature extractor: in the **1st**-hierarchy, we build multiple separate fully-connected multi-layer perceptron (MLP) neural networks sharing the same network architecture, and each MLP extracts the intra-modal latent feature of an independent modality of clinical data; in the **2nd**-hierarchy, the extracted latent features of all modalities are concatenated, then input to another similar MLP for cross-modal feature fusion.

Extensive experiments are conducted on a dataset consisting of 216 patients diagnosed with COVID-19, which has passed the review of the medical ethics committee and can be used for research purposes only. These patients were admitted to the Public Health Medical Center in Chongqing, China, and received intensive medical care. The experimental results of the prognostic study show the advantage of sequential stage-wise learning of MMDL over conventional single-stage learning. In addition, the results also prove that MMDL with multimodal inputs can surpass the reduced model with any single-modal clinical data input by a large margin, particularly for the severe group in disease severity assessment and the mild-to-severe incidence group in the disease progression prediction.

## 2. Dataset description

### 2.1. Patient demographics

We retrospectively review the medical records of 216 patients with COVID-19 who were admitted to the Public Health Center in Chongqing, China from January 24, 2020, to February 16, 2020. These patients were admitted fulfilling the following criteria: (1) tested positive with two consecutive nucleic acid tests; (2) showed distinct characteristics of pneumonia in CT images.

**Figure 1:**
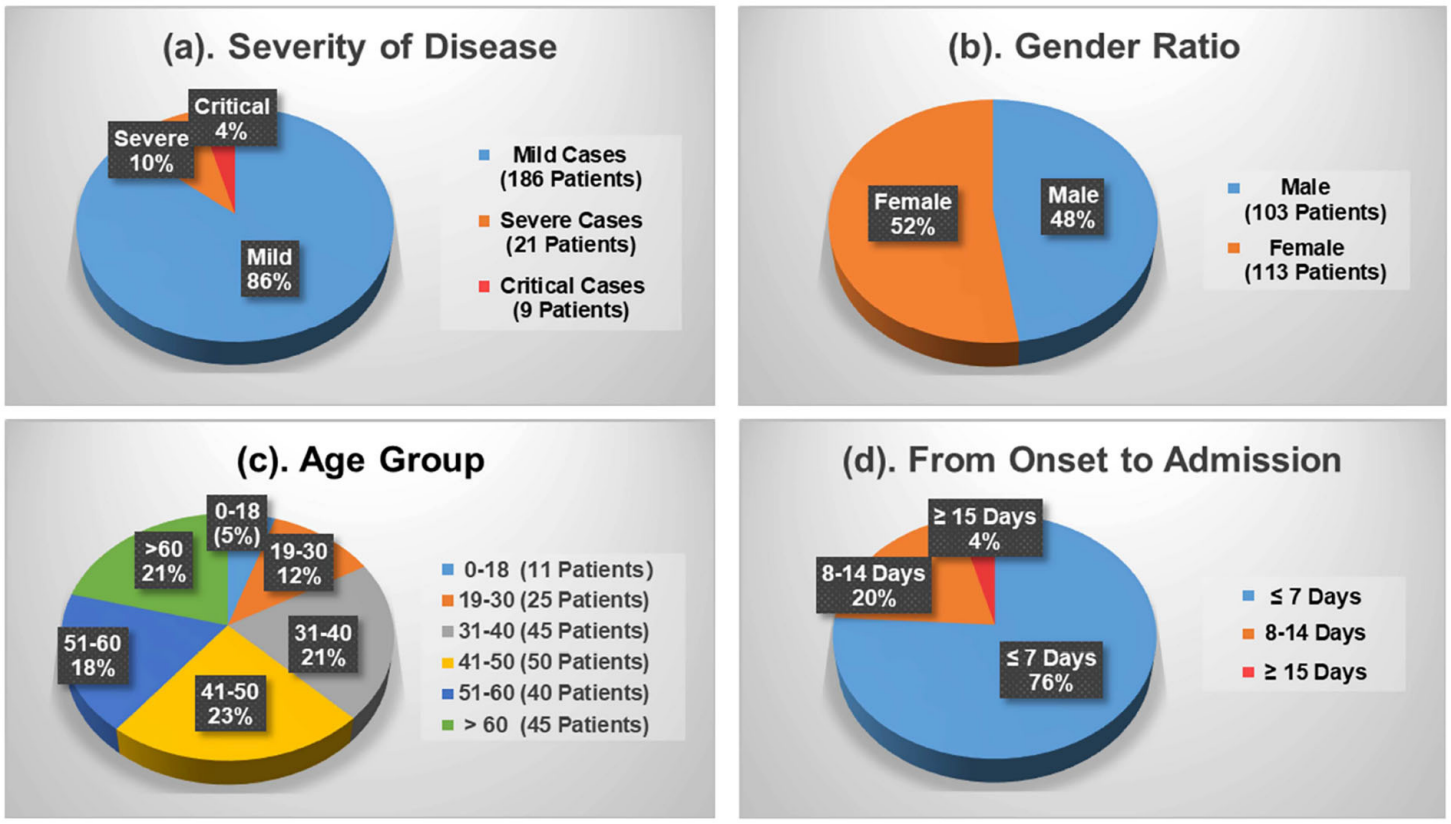
Demographics of the COVID-19 patients contained in the dataset. **(a)** Severity of disease. **(b)** Gender ratio. **(c)** Age group. **(d)** From onset to admission.

Figure 1 shows the demographics of these admitted patients that 103 cases (47.69%) out of the total number were male patients while female patients occupied 52.31% (113 cases). Depending upon the patient's severity of symptoms, 186 cases (86.11%) and 30 cases (13.89%) are diagnosed with mild and severe symptoms, respectively. By age, patients aged between 41 and 50 are the largest group with 50 cases (23.15%), which is followed by the group 31–40 and the group 60+ accounting for 20.83% (45 cases) each. Patients under the age of 18 and aged 19–30 only make up 5.09% (9 cases) and 11.57% (25 cases), respectively. Moreover, Figure 1d shows the average duration of onset of symptoms to hospital admission. A total of 76 and 20% of the patients were admitted in the first and the second week, respectively, since the onset of the disease. The remaining 4% of the patients developed symptoms after 2 weeks.

**Figure 2:**
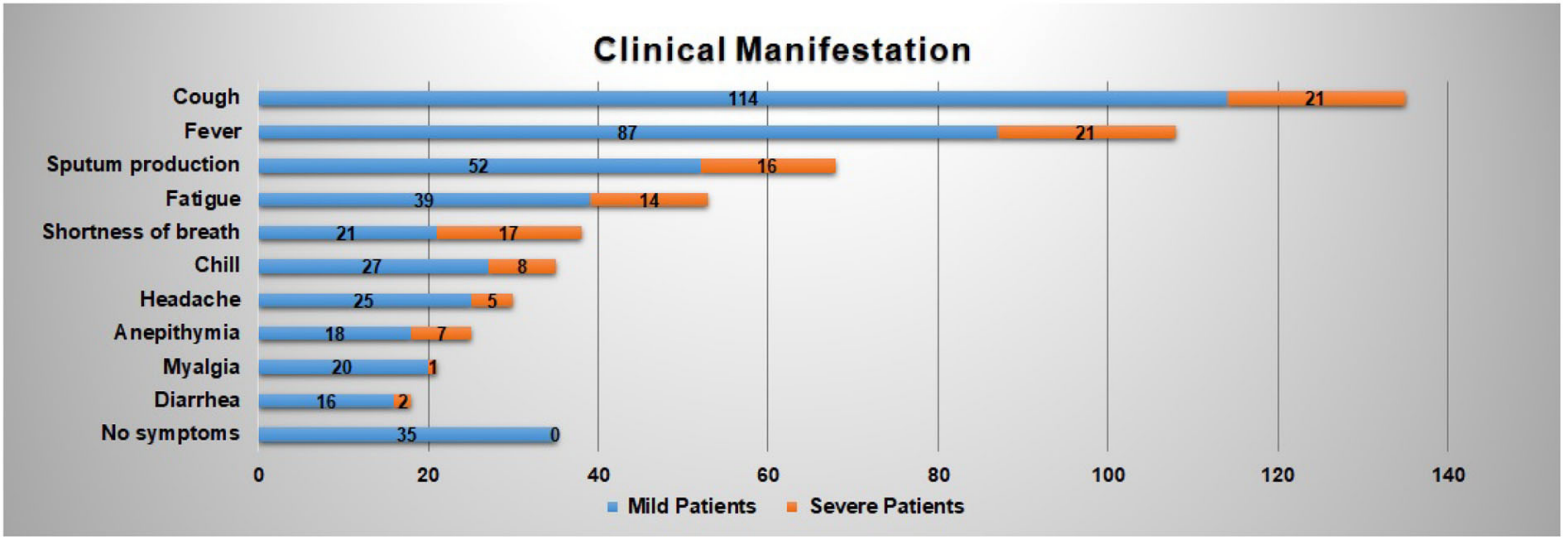
Clinical manifestation of the COVID-19 patients contained in the dataset.

Figure 2 reveals the top 10 clinical manifestations of the 216 patients with COVID-19 infections. As it shows, cough [135 cases (62.50%)], fever [108 cases (50.00%)], and expectoration [68 cases (31.48%)] are reported as the most typical symptoms. It records 53 cases (24.54%) of fatigue and 38 cases (17.59%) of shortness of breath, which are another two common clinical manifestations. In addition, about 35 patients show no symptoms on admission.

### 2.2. Multimodal clinical data

During the COVID-19 treatment, numerous patients' clinical data are produced including patients' vital signs, laboratory test results, CT image findings, medical experts' diagnoses, and corresponding treatment plans. Among these clinical data, laboratory test results comprise 11 different categories, which is termed “multimodal” in the context of big data and machine learning. Specifically, the 11 modalities are named blood test, flow cytometry, inflammation, liver function, renal function, blood lipids, glucose, electrolyte, myocardial zymogram and heart failure indicator, coagulation, and arterial blood gas. Each modality contains many laboratory test items. For example, the blood test modality consists of white blood cell count (WBC), red blood cell count (RBC), neutrocyte count (NEUT#), monocytes count (MONO#), lymphocyte count (LYMPH#), etc., and the inflammation modality contains erythrocyte sedimentation rate (ESR), C-reactive protein (CRP) and hypersensitive C-reactive protein (hs-CRP), and procalcitonin (PCT).

Table 1 describes the statistical results of the 11 modalities of laboratory tests of the mild group, the severe group, and the total population below:

**Table 1:** Characteristics of multimodal lab test results of the COVID-19 patients contained in the dataset.

** Characteristics**	**All patients**	**Mild patients**	**Severe patients**
	***N* = 216**	***N* = 186 (86.11%)**	***N* = 30 (13.89%)**
**Blood test**			
White blood cell (WBC), × 10^9^/*L*	6.03	5.32	6.47
Neutrophils (NEUT), × 10^9^/*L*	4.05	3.32	4.98
Lymphocyte (LYMPH), × 10^9^/*L*	1.34	1.43	0.86
Monocytes (MONO), × 10^9^/*L*	0.40	0.42	0.36
Eosinophils (EO), × 10^9^/*L*	0.06	0.06	0.06
Basophils (BASO), × 10^9^/*L*	0.02	0.02	0.02
Red blood cell (RBC), × 10^12^/*L*	4.20	4.25	4.00
Hemoglobin (HGB), *g*/*L*	128	130	124.3
Hematocrit (HCT), *L*/*L*	38.80	39.10	37.57
Mean corpuscular hemoglobin concentration (MCHC), *g*/*L*	331.6	332	331.2
Platelet (PLT), × 10^9^/*L*	229	216	244.8
Mean platelet volume (MPV), *fL*	9.40	9.30	9.62
Platelet hematocrit (PCT), (%)	0.21	0.20	0.23
**Flow cytometry**			
Absolute CD3+ T lymphocyte, *cells*/μ*L*	729	829	461
Absolute CD4+ T lymphocyte, *cells*/μ*L*	420	451	259
Absolute CD8+ T lymphocyte, *cells*/μ*L*	281	316	145
CD4+ / CD8+ ratio	1.43	1.38	1.47
**Inflammation**			
Erythrocyte sedimentation rate (ESR), *mm*/*h*	46.6	38.2	71.3
C-reactive protein (CRP), *mg*/*L*	26.2	17.3	65
Hypersensitive C-reactive protein (hsCRP), *mg*/*L*	32.5	21.9	71.1
Procalcitonin (PCT), *ng*/*L*	0.096	0.040	0.331
**Liver function**			
Prealbumin (PA), μ*g*/*dL*	221	227	200
α-*L*-Fucosidase (AFU), *U*/*L*	27.8	27.4	29.7
Alanine aminotransferase (ALT), *U*/*L*	34	28.1	59.3
Aspartate aminotransferase (AST), *U*/*L*	27.8	24.6	41.5
Alkaline phosphatase (ALP), *IU*/*L*	58.9	57.2	66.1
Gamma-glutamyltransferase (GGT), *IU*/*L*	43.3	32.2	90.7
Lactate dehydrogenase (LDH), *IU*/*L*	235	209	343
Total protein (TP), *g*/*L*	66.7	67.3	64.5
Albumin (ALB), *g*/*L*	40.2	41.2	35.5
Globulin (GLB), *g*/*L*	27.3	26.8	29.5
A/G Ratio	1.40	1.50	1.24
Total bilirubin (TBIL), μ*mol*/*L*	15.6	15.8	15.1
Total bile acid (TBA), μ*mol*/*L*	3.00	3.10	2.34
**Renal function**			
UREA, *mmol*/*L*	3.83	3.71	4.10
Creatinine (CREA), μ*mol*/*L*	66.9	67.2	65.9
Uric acid (UA), μ*mol*/*L*	303	320	231
Beta 2-microglobulin (β2−*M*), *mg*/*L*	2.19	2.16	2.38
Cystatin C (CysC), *mg*/*L*	0.99	0.96	1.16
**Glucose modality**			
Glucose (hexokinase (HK) method), *mmol*/*L*	6.41	6.11	7.73
**Blood lipids**			
Triglyceride (TG), *mmol*/*L*	2.23	2.11	2.69
Total cholesterol (CHOL), *mmol*/*L*	4.39	4.28	4.26
High-density lipoprotein (HDL), *mmol*/*L*	1.02	1.03	0.97
Low-density lipoprotein (LDL), *mmol*/*L*	2.47	2.42	2.38
**Electrolyte**			
Potassium (K), *mmol*/*L*	4.21	4.24	4.00
Sodium (NA), *mmol*/*L*	138.2	138.4	137.8
Chlorine (CL), *mmol*/*L*	102.7	103.0	101.5
Calcium (CA), *mmol*/*L*	2.24	2.26	2.13
Phosphorus (P), *mmol*/*L*	1.08	1.11	0.98
Magnesium (MG), *mmol*/*L*	0.89	0.88	0.93
**Coagulation function**			
Prothrombin time (PT), *seconds*	11.77	11.76	11.81
International normalized ratio (INR)	1.00	1.01	0.96
Activated partial thromboplastin time (APTT), *seconds*	38.50	38.40	39.25
Thrombin time (TT), *seconds*	14.90	14.70	15.15
Fibrinogen (FIB), *g*/*L*	4.25	4.20	4.62
D-dimer, *mg*/*L*	0.68	0.44	1.64
**Myocardial zymogram & Heart failure**			
Adenosine deaminase (ADA), *U*/*L*	14.10	13.85	14.36
Creatine kinase (CK), *U*/*L*	100	88	151
α-Hydroxybutyrate dehydrogenase (α-HBDH), *IU*/*L*	179	160	262
5'-Nucleotidase (5'-NT), *U*/*L*	4.38	3.94	6.25
Cholinesterase (CHE), *U*/*L*	7960	8409	6699
**Arterial blood gas**			
Arterial blood pH	7.42	7.41	7.45
Partial pressure of oxygen (PaO2), *mmHg*	85	85	86
Partial pressure of carbon dioxide (PaCO2), *mmHg*	41	41	40
Bicarbonate (HCO3-), *mEq*/*L*	26.10	26.00	26.95
Oxygen saturation (SaO2), (%)	96.90%	97.30%	96.15%

## 3. Summary of notations

All the notations used in this paper are summarized below:

**Table:** 

$F_{\text{Θ}}$	MMDL model for disease severity assessment with network parameters Θ;
$F_{\text{Φ}}$	MMDL model for disease progression prediction with network parameters Φ;
**X** ^(**n**)^	A matrix. $X^{\left(n\right)}\text{​}=\text{​}[X_{1}^{\left(n\right)},...,X_{k}^{\left(n\right)},...,X_{K}^{\left(n\right)}]$ is the input multimodal clinical data of the *n*-th stage;
$X_{k}^{\left(n\right)}$	A vector. The *k*-th modality clinical input data of the *n*-th stage;
$X_{k}^{\prime (n)}$	A vector. The extracted latent feature of *k*-th modality of the *n*-th stage;
$X_{\text{CAT}}^{\left(n\right)}$	A matrix. $X_{\text{CAT}}^{\left(n\right)}=[X_{1}^{\prime (n)},...,X_{k}^{\prime (n)},...,X_{K}^{\prime (n)}]$ is the concatenation of the extracted intra-modal latent features of all different modalities of the *n*-th stage;
*N*	A scalar. The total rounds (stages) of the performed clinical examination and lab tests;
*K*	A scalar. The total number of input modalities, *K* = 13, including the patient demographics, clinical manifestation and laboratory test results (e.g., blood test, inflammation, liver function, renal function, blood lipids, etc.);
**D**	A vector. The extracted latent feature of patient demographics modality;
**Z** ^(*n*)^	A vector. The extracted cross-modal feature representation of the *n*-th round clinical manifestation modality and other 11 laboratory test modalities;
**S** ^(*n*)^	A vector. The intermediate learning outcome of the *n*-th stage;
**W**_**D**_	A matrix. The weighting matrix multiplying with **D**;
${\text{W}}_{\text{Z}}^{\left(n\right)}$	A matrix. The weighting matrix multiplying with **Z**^(*n*)^;
${\text{W}}_{\text{S}}^{\left(n\right)}$	A matrix. The weighting matrix multiplying with **S**^(*n*)^;
${\text{b}}_{\text{S}}^{\left(n\right)}$	A vector. The bias vector added to the computed results at stage *n*;
α	A scalar. α ∈ [0, 1] is a decay factor, by multiplying with which the learning outcome of the previous stage **S**^(*n*)^ is attenuated every round;
$\bar{Y}$	A vector. The output vector for computing $\bar{y}$;
$\bar{y}$	A scalar. The obtained result of either disease severity assessment ($\bar{y}\in \left\{mild,severe\right\}$) or disease progression prediction ($\bar{y}\in \left\{not\text{−}develop\text{−}severe,develop\text{−}severe\right\}$);
*y*	A scalar. The corresponding ground truth label;
*MLP*	The fully connected multilayer perceptron neural network;
*ReLU*	The rectified linear unit activation function;
*Softmax*	The softmax multi-class classifier;
$L_{\text{cross-entropy}}$	The cross-entropy loss function.

## 4. Problem formulation

Given patients infected by COVID-19 take *N* rounds of clinical examination and laboratory tests in total during the treatment. In each round, *K* different modalities of clinical data (e.g., clinical manifestation, blood test, inflammation, liver function, etc.) are collected for disease assessment and prediction. Take the *n*-th round as an example, the notation **X**^(*n*)^ is used to denote the stage-wise multimodal input:


(1) $$X^{\left(n\right)}=[X_{1}^{\left(n\right)},X_{2}^{\left(n\right)},\ldots ,X_{k}^{\left(n\right)},\ldots ,X_{K}^{\left(n\right)}],$$

where 1 ≤ *n* ≤ *N* and 1 ≤ *k* ≤ *K*.

By leveraging the stage-wise multimodal clinical data, **X**^(1)^, **X**^(2)^, …, **X**^(*n*)^, …, **X**^(*N*)^, our goal is to develop a model F to: (1) assess the disease severity of patients diagnosed with COVID-19, and (2) forecast mild cases who have a high risk of progressing to critical illness. The two tasks share the same network architecture but are trained separately with two different sets of network parameters.

Mathematically, in the disease severity assessment task, it can be expressed as:


(2) $${\bar{y}}_{\bar{y}\in \{mild,\;severe\}}={ℱ}_{\text{Θ}}(X^{\left(1\right)},X^{\left(2\right)},\ldots ,X^{\left(n\right)},\ldots ,X^{\left(N\right)}|\text{Θ}),$$

where $\bar{y}\in \left\{mild,severe\right\}$ is used to denote the obtained result of assessment, $F_{\Theta }$ represents the MMDL model with the parameter Θ that maps the multistage input to the output $\bar{y}$.

Similarly, in the disease progression prediction task, it can be written as:


(3) $$\begin{array}{l} {\bar{y}}_{\bar{y}\in \{not\text{​}-\text{​}develop\text{​}-\text{​}severe,\;develop\text{​}-\text{​}severe\}}=\\ {ℱ}_{\Phi }(X^{\left(1\right)},X^{\left(2\right)},\ldots ,X^{\left(n\right)},\ldots ,X^{\left(N\right)}|\text{Φ}), \end{array}$$

Likewise, ${\bar{y}}_{\bar{y}\in \left\{not\text{−-}develop\text{−-}severe,develop\text{−-}severe\right\}}$ is the predicted results and $F_{\Phi }$ is the corresponding prediction model with network parameters Φ.

## 5. Multistage multimodal deep learning model

In this section, we introduce the multistage, multimodal deep learning (MMDL) model in detail. We first illustrate the sequential stage-wise learning framework, then present the feature extraction of the multimodal clinical data at each stage, and finally come to the end-to-end training of MMDL.

### 5.1. Sequential stage-wise learning

Sequential stage-wise learning and sequence prediction share some common ground, although they are different in some respects. The sequence-to-sequence (seq2seq) model (29, 30) is one of the classical benchmarks in contextual sequence prediction. It transforms one sequence into another sequence, and the context of the decoded sentence is based on the output from its previous contexts.

**Figure 3:**
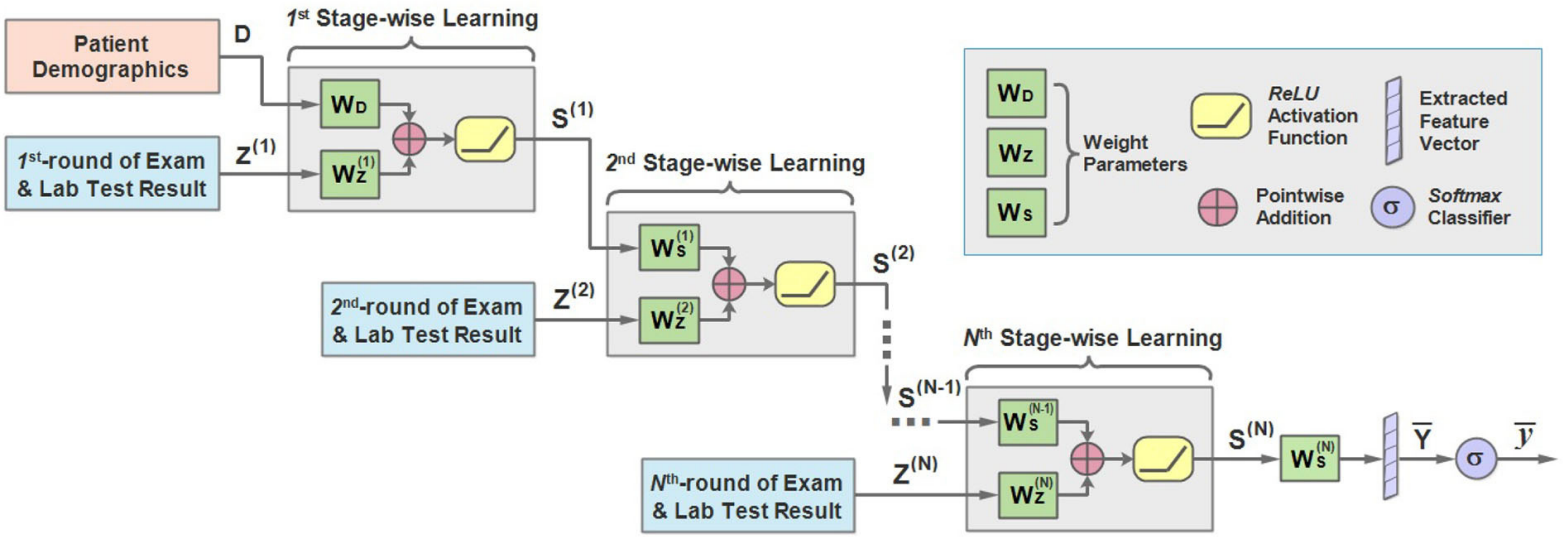
An illustration of the architecture of the sequential stage-wise learning of the MMDL model.

Motivated by this, we propose the sequential stage-wise learning architecture of MMDL, which is illustrated in Figure 3. As we can see, it meticulously joins the extracted cross-modal latent feature of the previous stage and the current stage, then concatenates the result with the extracted multimodal feature of the next stage sequentially for further processing.

In the first stage, the model takes the patient demographics **D** and the initial examination and laboratory test results **Z**^(1)^ when admitted to the hospital as the model input. It should be noted that **D** is the extracted latent feature of the patient demographics modality only, and **Z** is the merged multimodal feature representation across all different modalities of lab test results. How **Z** is extracted and merged will be justified in the next subsection in detail.

**D** and **Z** first multiply with **W**_**D**_ and ${\text{W}}_{\text{Z}}^{\left(1\right)}$ respectively, and add together, then pass through the *ReLU* activation function.


(4) $$S^{\left(1\right)}\;=ReLU(W_{D}D+W_{Z}^{\left(1\right)}Z^{\left(1\right)}+b_{S}^{\left(1\right)}),$$

where **S**^(1)^ is the output of the first stage. **W**_**D**_ and ${\text{W}}_{\text{Z}}^{\left(1\right)}$ are the weight matrices, and ${\text{b}}_{\text{S}}^{\left(1\right)}$ is the bias term. *ReLU* is short for rectified linear units (31), which can be expressed as *ReLU*(*x*) = *max*(0, *x*) and is a non-linear activation function.

Then ${\text{b}}_{\text{S}}^{\left(1\right)}$ multiplies with the weight ${\text{W}}_{\text{s}}^{\left(1\right)}$ accordingly and adds to the result of the multiplication of **Z**^(2)^ and ${\text{W}}_{\text{Z}}^{\left(2\right)}$. Similarly, we can derive the expression of the learning process of the second stage:


(5) $$S^{\left(2\right)}\;=ReLU(W_{Z}^{\left(2\right)}Z^{\left(2\right)}+\alpha W_{S}^{\left(1\right)}S^{\left(1\right)}+b_{S}^{\left(2\right)}),$$

in which **Z**^(2)^ is the extracted cross-modal latent feature of the second stage and ${\text{W}}_{\text{Z}}^{\left(2\right)}$ is the corresponding weight. It should be mentioned that $W_{\text{S}}^{\left(1\right)}{\text{S}}^{\left(1\right)}$ is attenuated by multiplying with a decay factor α ∈ [0, 1] because the new round of test results has a direct impact on the predicted results while the influence of the test result obtained long ago weakens as time passes.

Note that the modality of patient demographics is incorporated into the model in the initial stage only since the patient demographics modality contains patients' basic information, like gender and age, that does not change in every round of test.

For the *n*-th stage (2 ≤ *n* ≤ *N*), a more general form can be written as:


(6) $$S^{\left(n\right)}\;=ReLU(W_{Z}^{\left(n\right)}Z^{\left(n\right)}+\alpha W_{S}^{\left(n-1\right)}S^{\left(n-1\right)}+b_{S}^{\left(n\right)}),$$

Finally, the learned representation of the last stage ${\text{W}}_{\text{S}}^{\left(N\right)}$ is further fused to get the output vector $\bar{Y}$:


(7) $$\bar{Y}=W_{S}^{\left(N\right)}S^{\left(N\right)}+b_{S}^{\prime },$$

and pass it through a multi-class Softmax classifier to get the predicted outcome $\bar{y}$.


(8) $$\bar{y}=Softmax(\bar{Y}),$$

$\bar{y}$ is the output scalar, which is either *mild* or *severe* in the first task and *not*−*develop*−*severe* or *develop*−*severe* in the second.

### 5.2. Multimodal feature extraction

**Figure 4:**
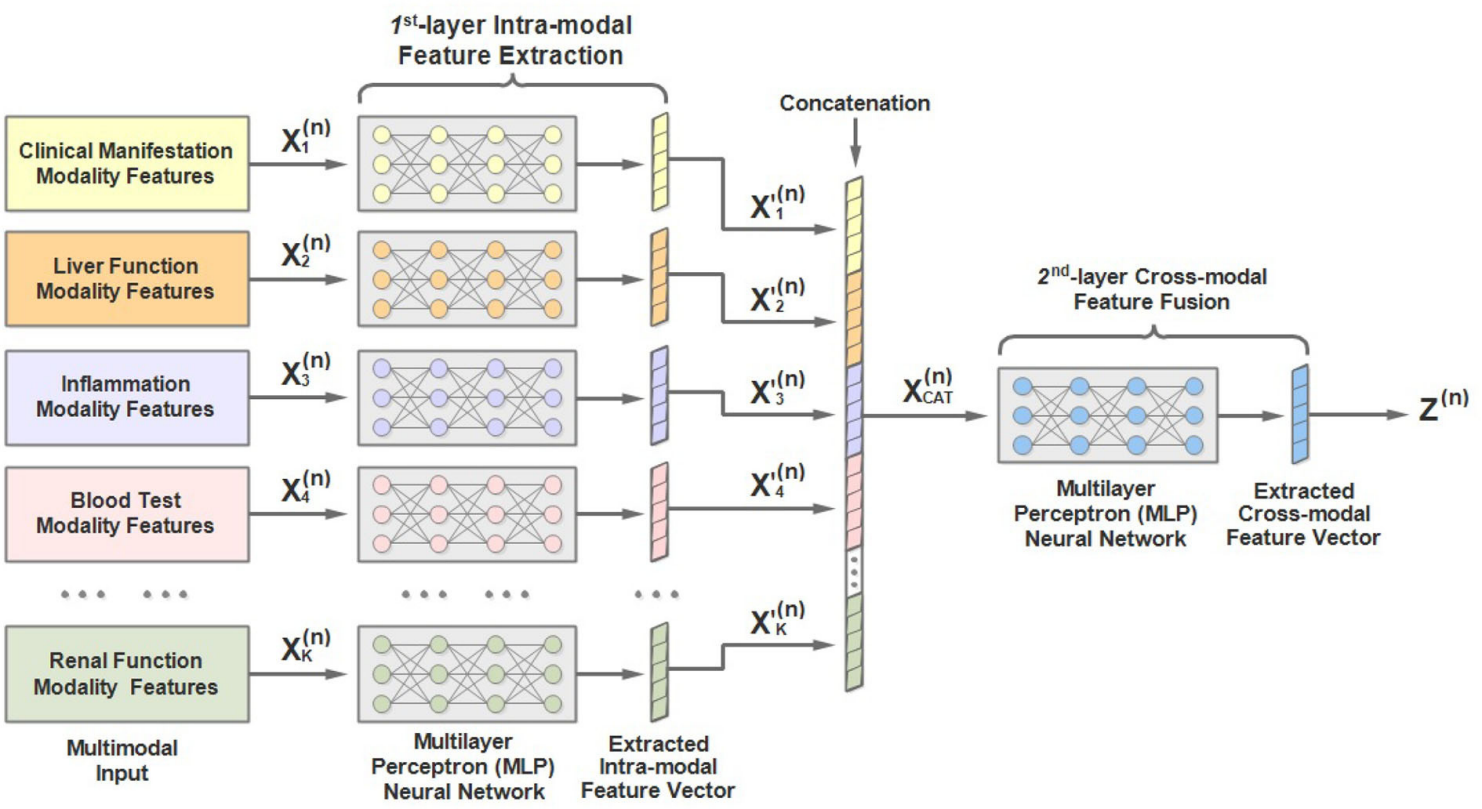
An illustration of the architecture of multimodal feature extractor of the MMDL model.

As mentioned above, the input to each stage is multimodal lab test results. To address the characteristics of the multimodal input data, we would like to introduce a two-layer multimodal feature extractor conceived by us and the architecture of which is shown in Figure 4. As we can see, the **1st**-hierarchy aims to perform intra-modal feature learning and extraction, while the **2nd**-hierarchy attempts to perform cross-modal feature fusion.

Concretely, in the **1st** hierarchy, we build up *K* (*K* = 13) independent multilayer perceptron (MLP) neural networks. Each MLP is responsible for extracting the latent feature of a separate input modality, including the patient demographics, clinical manifestation, and 11 other modalities of laboratory test data.

For example, the extracted feature of the *k*-th modality of the *n*-th round test can be expressed as:


(9) $$X_{k}^{\prime (n)}=MLP(X_{k}^{\left(n\right)}),$$

where $X_{k}^{\prime (n)}$ denotes the extracted latent feature vector of the *k*-th modality (1 ≤ *k* ≤ *K*), and *MLP* represents the multi-layer fully-connected neural network.

Afterwards, the extracted intra-modal feature vectors of all modalities $X_{1}^{\prime (n)},X_{2}^{\prime (n)},\ldots ,X_{k}^{\prime (n)},\ldots ,X_{K}^{\prime (n)}$ are concatenated:


(10) $$X_{CAT}^{\left(n\right)}=[X_{1}^{\prime (n)},X_{2}^{\prime (n)},\ldots ,X_{k}^{\prime (n)},\ldots ,X_{K}^{\prime (n)}].$$

Finally, the concatenated feature vectors $X_{\text{CAT}}^{\left(n\right)}$ are further processed by another *MLP* to obtain the fused cross-modal feature representation **Z**^(*n*)^, which is then taken as the input of the *n*-th stage of MMDL:


(11) $$Z^{\left(n\right)}=MLP(X_{CAT}^{\left(n\right)}),$$

### 5.3. Model training

Before training the model, we have to define the loss function in the first place, which gives the learning objective during the training process. The loss function compares the difference between the predicted results $\bar{y}$ and the ground truth labels *y* given by medical experts, and a smaller value of L means the model's performance is better. Either the patient disease assessment or the disease progression prediction can be regarded as a classification problem, hence we choose cross-entropy as the loss function, which is widely used in multi-class classification problems:


(12) $${ℒ}_{\text{ cross-entropy}}=-\sum_{j}y_{j}\cdot \log(p(\bar{y}\text{​}=\text{​}j)),$$

where *j* represents the predicted class, and *j* = {*mild, severe*} for the disease severity assessment and *j* = {*not* − *develop* − *severe, develop*−*severe*} for disease progression prediction. $p\left(\hat{\text{y}}=j\right)$ is the predicted probability of the class *j* using *Softmax*, i.e.,


(13) $$p(\bar{y}=j)=Softmax(Y_{j})=\frac{e^{Y_{j}}}{\sum_{i=1}^{\#class}e^{Y_{i}}}.$$

The notation *#class* represents the number of classes, and *#class* = 2 in our settings because there are two results for both the assessment and the prediction tasks.

During the training process, the end-to-end supervised learning is used to train MMDL. Adam optimizer is adopted to backpropagate the calculated loss to the input layer of the model, and all network parameters (weights and biases) are updated through iterative optimization. MMDL is trained two times separately to learn two different sets of network parameters, i.e., Θ and Φ, one for the disease severity assessment and the other for the disease progression prediction.

## 6. Experiments

In this section, we will present the experimental part of MMDL in detail. We first introduce how we set up the experiment, then the evaluation metrics, and finally present the comparison results for both tasks.

### 6.1. Experiment setup

In the experiment, we first assess the severity of illness of patients using different numbers of consecutive stages of multimodal inputs (*#Multistage Input*), then forecast whether patients with mild symptoms will progress to severe illness or not with different prediction steps (*Prediction Step*). To start with, we assess the severity of illness of patients using the initial exam and lab test data on admission, then identify patients diagnosed with mild symptoms who are prone to develop severe symptoms. Subsequently, we extend it to the scenario of the diesese severity assessment using multistage input, i.e., use multiple successive rounds of clinical test data to assess the disease severity.

In addition, in view of the limited samples contained in the dataset, 10-fold cross validation is adopted, that is, in each round of training, 10% of the cases are randomly selected for testing and the remaining 90% cases are used for training, while in another round, another 10% cases are selected as the test set.

### 6.2. Evaluation metric

We use a group of evaluation metrics to evaluate the classification performance of MMDL, including accuracy, error rate, precision, recall, and F1 score, which are computed as follows:


(14) $$\{\begin{array}{l} Accuracy=\frac{TP+TN}{TP+TN+FP+FN},\\ Error\;Rate=1-Accuracy,\\ Precision=\frac{TP}{TP+FP},\\ Recall=\frac{TP}{TP+FN},\\ F1=\frac{2}{\frac{1}{Precision}+\frac{1}{Recall}}=\frac{2\times TP}{2\times TP+FP+FN}, \end{array}$$

where TP, FP, TN, and FN represent True Positive, False Positive, True Negative, and False Negative samples, respectively. The higher the value obtained, the better performance is achieved for all evaluation metrics but the error rate.

### 6.3. Results

#### 6.3.1. Disease severity assessment with different numbers of multistage input

To show the advantage of learning with multistage data, we compare the performance of MMDL using a single stage's inputs (i.e, *#Multistage Input* = 1) and multiple successive stages' inputs (i.e, *#Multistage Input*>1) on disease severity assessment (i.e., *Prediction Step* = 0).

**Figure 5:**
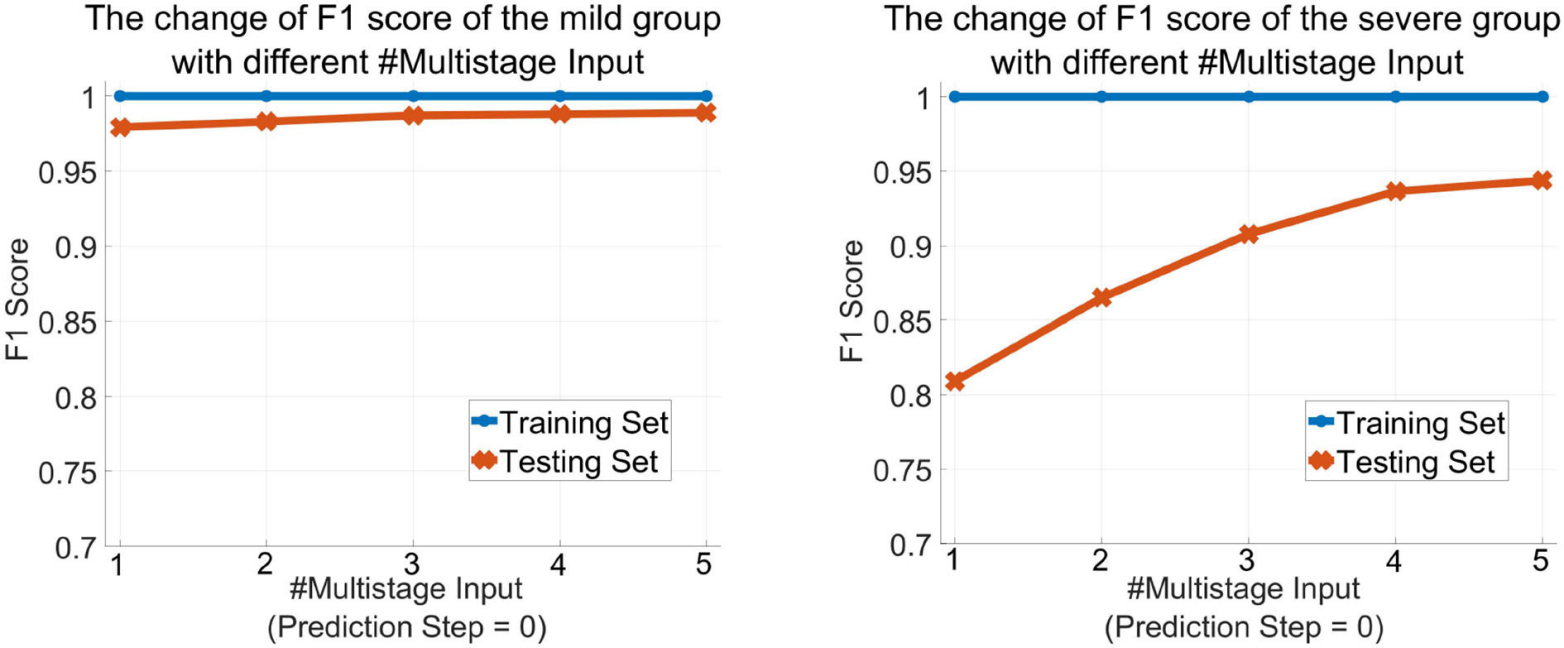
The change of the F1 score of MMDL model as the increase in the number of multistage inputs of the mild group **(left)** and the severe group **(right)**.

Table 2 shows the obtained results. Accuracy is the proportion of the correctly classified samples (i.e., TP + TN) to the total number of samples, so the mild and the severe groups have the same accuracy, which increases from 96.26% using the current stage inputs only to 98.10% using five consecutive stages' inputs. Precision is the correct predictions (i.e., TP) out of all patients predicted to be infected (i.e., TP + FP), which grows from 97.52% with *#Multistage Input* = 1 to 98.69% with *#Multistage Input* = 5, respectively. Recall, which represents the percentage of truly predicted infections (i.e., TP) among all infections (i.e., TP + FN), goes from 98.33 to 99.60%. F1 is the harmonic mean of precision and recall, which is a more balanced evaluation metric to reflect the overall classification results. Moreover, we plot the curve depicting the change of F1 as the increase of the numbers of used multistage input in Figure 5. We can see that in the training phase, the obtained results are all 100% for both the mild and severe groups, which reveals that MMDL fits the training set perfectly. In the testing phase, the F1 score of the mild and the severe groups increases from 0.9792 and 0.8091 by simply taking a single stage's inputs to 0.9890 and 0.9436 by considering all five successive stages' multimodal data, respectively.

**Table 2:** Performance comparison of MMDL model with different numbers of multistage inputs.

	**#Multistage**	**Prediction**	**Accuracy**	**Error Rate**	**Precision**	**Recall**	**F1 Score**
	**Input data**	**Step**					
Mild group	1	0	96.26%	3.73%	97.52%	98.33%	0.9792
	2	0	96.98%	3.01%	97.74%	98.86%	0.9830
	3	0	97.74%	2.25%	98.03%	99.40%	0.9871
	4	0	98.09%	1.90%	98.60%	99.50%	0.9887
	5	0	98.10%	1.89%	98.69%	99.60%	0.9890
Severe group	1	0	96.26%	3.73%	84.12%	77.94%	0.8091
	2	0	96.98%	3.01%	90.56%	82.75%	0.8648
	3	0	97.74%	2.25%	95.52%	86.48%	0.9078
	4	0	98.09%	1.90%	95.16%	92.18%	0.9365
	5	0	98.10%	1.89%	95.71%	93.05%	0.9436

#### 6.3.2. Prediction of disease progression with different prediction steps

In this subsection, we would like to forecast progression from mild to severe COVID-19. First, it gives a brief introduction to the labels of the dataset. Medical experts assess patients' status after every round of the exam and lab test, which is treated as the ground truth labels of the prediction task of that stage. We set *Prediction Step* = 1 if we want to predict the patient's condition after the next round's test, and *Prediction Step* = 4 if we predict the patient's situation four stages ahead.

**Figure 6:**
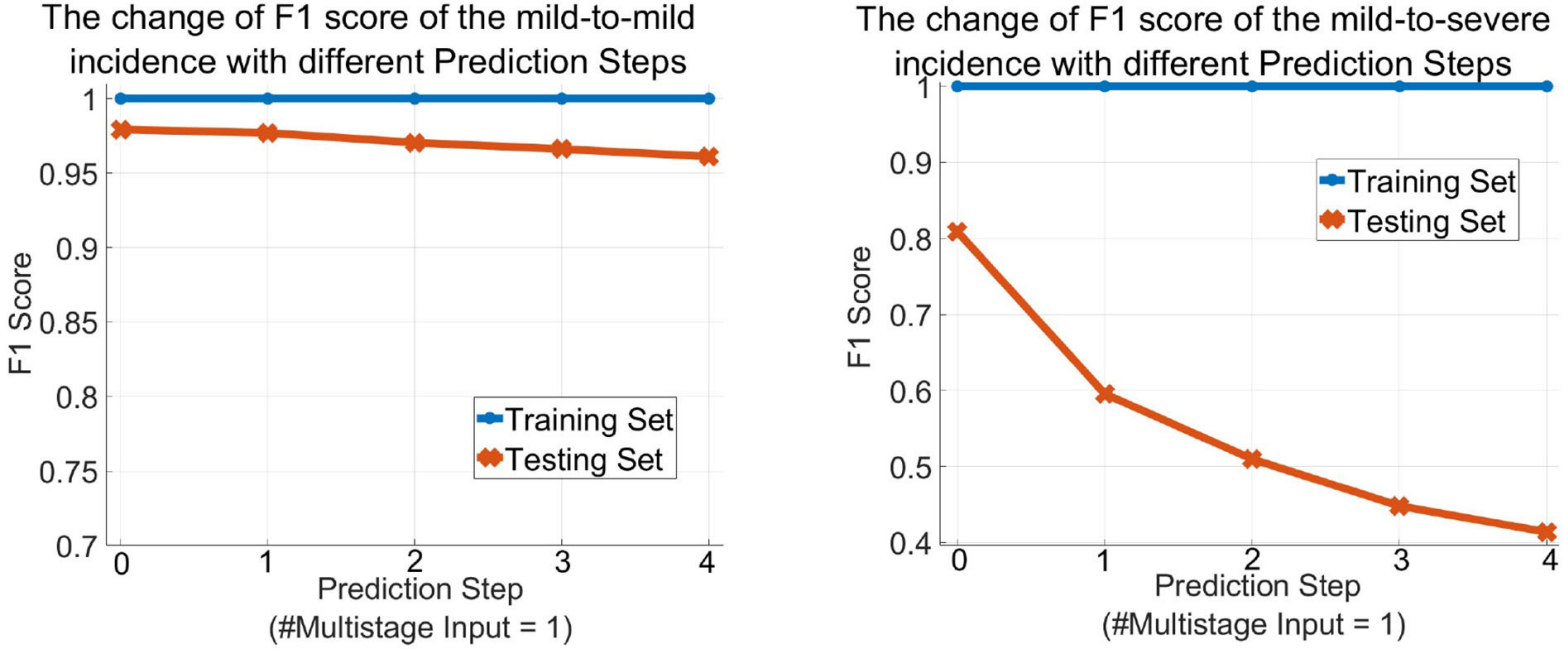
The change of the F1 score of the MMDL model as the increase of the prediction step of the mild group **(left)** and the severe group **(right)**.

Table 3 describes the predicted results as the increase of the *PredictionStep*. The accuracy, precision, and recall of the mild group are 96.26, 97.52, and 98.33%, respectively, when *Prediction Step* = 0, then gradually decrease to 93.44, 95.74, and 97.12% when predicting the state of the illness of patients four stages away from now (*Prediction Step* = 4). For the severe group, these figures start from 96.26, 84.12, and 77.94%, then drop rapidly and finally stop at 93.44, 48, and 36.36%, respectively. Figure 6, left, right plot the curves of the F1 score of the mild-to-mild and mild-to-severe progressions as the increase of *Prediction Step*. In the testing phase, the F1 of the mild-to-mild incidence decreases from 0.9792 (*Prediction Step* = 0) to 0.9653 (*Prediction Step* = 4) gradually. But the situation worsens when predicting progression from mild to severe COVID-19 that F1 begins at 0.8091 (*Prediction Step* = 0), then declines dramatically to 0.5957 (*Prediction Step* = 1) and 0.5098 (*Prediction Step* = 2), then continues to decrease and finally stops at 0.4137 for *Prediction Step* = 4.

**Table 3:** Performance comparison of MMDL model with different prediction step.

	**#Multistage**	**Prediction**	**Accuracy**	**Error Rate**	**Precision**	**Recall**	**F1 Score**
	**Input data**	**Step**					
Mild group	1	0	96.26%	3.73%	97.52%	98.33%	0.9792
	1	1	95.63%	4.30%	96.86%	98.52%	0.9769
	1	2	94.43%	5.50%	96.25%	97.85%	0.9704
	1	3	93.61%	6.30%	96.00%	97.22%	0.9661
	1	4	93.44%	6.55%	95.74%	97.12%	0.9653
Severe group	1	0	96.26%	3.73%	84.12%	77.94%	0.8091
	1	1	95.63%	4.30%	70.00%	51.85%	0.5957
	1	2	94.43%	5.50%	59.09%	44.82%	0.5098
	1	3	93.61%	6.30%	50.00%	40.62%	0.4482
	1	4	93.44%	6.55%	48.00%	36.36%	0.4137

#### 6.3.3. The ROC and AUC of disease severity assessment and prediction of disease progression

**Figure 7:**
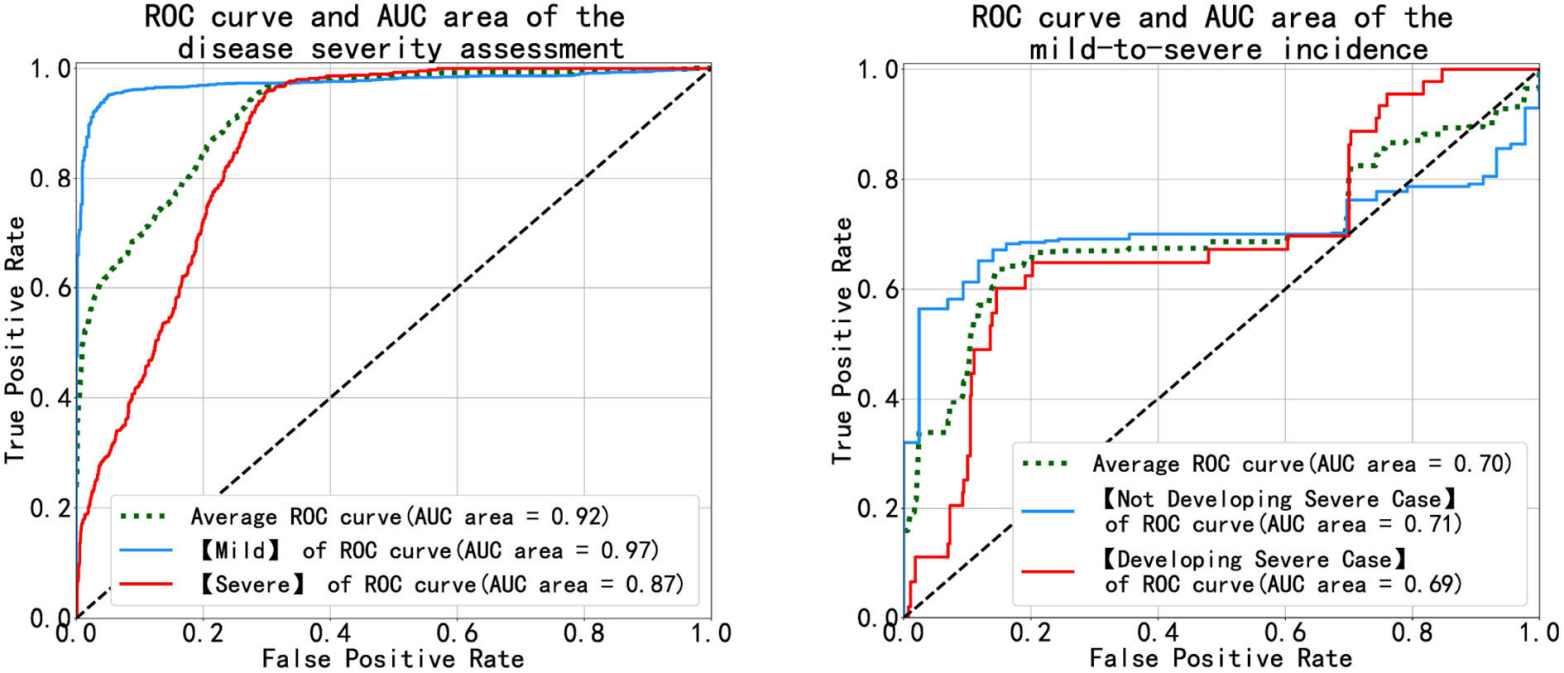
Diagrams of the ROC and AUC of the disease severity assessment **(left)** and the disease progression prediction **(right)**.

Figure 7 shows the receiver operating characteristic (ROC) and the area under the curve (AUC) of disease severity assessment (left) with *#Multistage Input* = 1 and *Prediction Step* = 0 and prediction of disease progression (right) with *#Multistage Input* = 1 and *Prediction Step* = 4. An ROC curve is a graph showing the performance of a classification model at different classification thresholds. The *x*-axis is FPR (False Positive Rate) which is calculated as $FPR=\frac{FP}{FP+TN}$, and the *y*-axis is TRP (True Positive Rate), also known as recall, which is computed as $TRP=\frac{TP}{TP+FN}$. Lowering the classification threshold classifies more items as positive, thus increasing both False Positives (FP) and True Positives (TP).

As Figure 7 (left) illustrates, the blue and red curves represent the ROC of the mild group and the severe group, respectively, and the green curve is the arithmetical average of them. The blue curve approaches the top-left corner, which means the MMDL model is a good classifier for distinguishing mild cases out of all infections. The classification result of severe cases is worse than that of the mild cases as the red curve is not as steep as the blue one in the beginning until FPR equals around 0.3, then the red curve approaches the blue one. In the right subfigure of Figure 7, it shows the ROC and AUC of patients' progression from mild to severe infection (denoted by the light blue curve) and of the ones that deteriorate (denoted by the red curve). As the figure shows, the achieved results of the MMDL model for predicting disease progression are not as good as those for assessing the patient's status. We can observe a distinct plateau region in Figure 7, right where both the blue and red curves do not go up. It means the MMDL model has difficulty distinguishing between the one developing and not developing severe symptoms when 0.2 ≤ *FPR* ≤ 0.7.

#### 6.3.4. The impact of multimodal deep learning on the performance of the MMDL model

To show the advantages of multimodal learning for feature extraction and fusion across different modalities of clinical data, we compare the performance of the complete MMDL model with multimodal inputs and the reduced models with separate single-modal inputs only in the testing phase.

**Figure 8:**
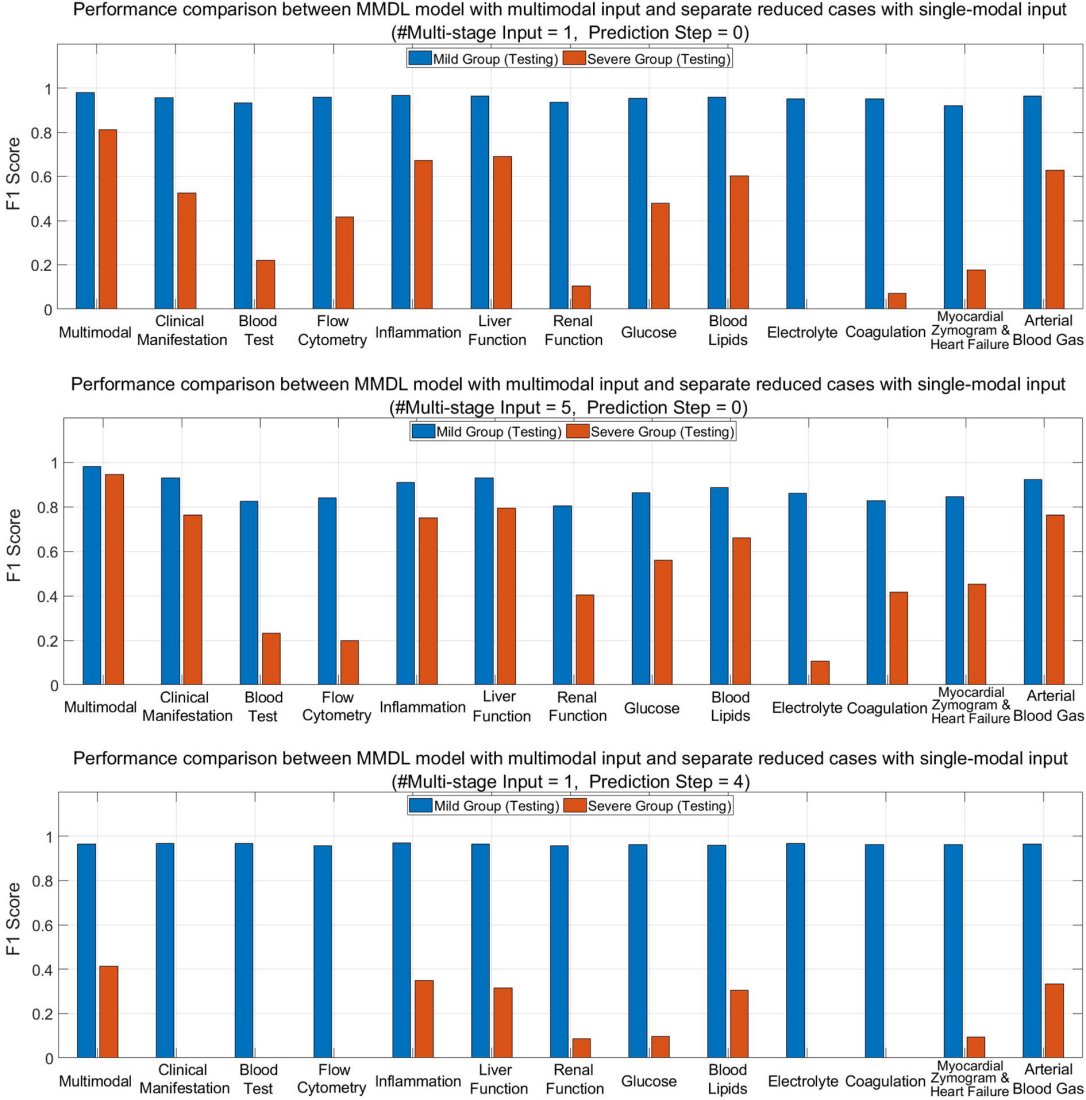
Performance comparison between MMDL model with multimodal input and reduced cases with separate single-modal inputs with different *#Multistage Input* and *Prediction Step*.

Figure 8 (upper) and (middle) compare MMDL using the latest round of exam and lab test results as the model input (*Multistage Input* = 1 and *PredictionStep* = 0) and leveraging the last five consecutive rounds of test data (*Multistage Input* = 5 and *PredictionStep* = 0). As we can see, the overall performance of the MMDL model for assessing the mild group exceeds that of the severe group by a large margin regardless of using single-stage or multistage inputs. Noted that the performance gain is limited for the mild group, particularly for *#Multistage Input* = 1, but significant for the severe group, which grows at least 15% for both *#Multistage Input* = 1 and *#Multistage Input* = 5.

Figure 8 (lower) depicts the bar chart of the F1 score for forecasting a patient's condition four stages away from now (*Prediction Step* = 4). From the diagram, we have the following observations: the achieved F1 score is good for mild-to-mild but, to some extent, terrible for mild-to-severe incidence prediction. It reveals the false-negative rate of the mild-to-severe incidence prediction is high, that is, samples are more prone to be classified as not developing severe symptoms. Nevertheless, MMDL predicting with multimodal inputs outperforms reduced models using any single-modal clinical data, which validates the superiority of multimodal learning. Furthermore, it is worth pointing out that among all modalities, inflammation, liver function, blood lipids, and arterial blood gas reach much higher F1 than any other modality. Hence, further explorations need to be conducted to discern the effective biomarkers within these modalities, which can be treated as signs to discriminate between mild cases developing and not developing severe symptoms.

## 7. Discussion

We notice that in the disease severity assessment task, MMDL's classification performance in the severe group is not as good as the mild group irrespective of using single-stage or multistage inputs. Similar observations are made in the disease progression prediction task as well that the prediction results of the mild-to-severe incidence fall far behind the mild-to-mild incidence. From our perspective, the reasons for this are twofold: (1) The patient samples contained in the dataset are quite limited for clinical data analysis and model development. We are only authorized to use these 200+ samples legally that pass the review of the ethics committees (RECs). However, ethics and compliance are extremely important in clinical research, and samples that fail to pass RECs are strictly forbidden to use; (2) What makes the situation even worse is the imbalanced distribution of patient samples (only 30 severe cases). As a result, it is insufficient to learn the characteristics of the patients of the severe group and the transition from mild to severe symptoms.

Besides the small sample learning, another challenge is that patient samples contained in the dataset were collected during the first wave of the pandemic, and the pre-trained model may no longer take effect as the virus has evolved to the Omicron variant in 2022. To address the challenge, we have deployed the prototype of MMDL in Chongqing Public Health Center, China, to validate the effectiveness of MMDL when facing new variants of COVID-19. Alongside model testing, we also collect new patient samples and attempt to train MMDL using new samples incrementally. Furthermore, to test the MMDL's availability in other chronic diseases, we are extending it to epilepsy prediction characterized by many follow-ups.

Another observation is that MMDL using multiple sequential stages' exam and lab test data outperforms the current stage's data in disease severity assessment. In particular, the latest three rounds' inputs dominate the assessment results, and history long ago has little influence on the model's output. Moreover, in predicting the disease progression, we can observe prediction results deteriorate as *Prediction Step* increases. It is because, according to our point of view, biomarkers show no significant abnormality to discriminate whether patients will turn for the worse in the distant future.

Also, experimental results validate multimodal feature extraction and fusion can provide complementary information to single-modal feature learning. Another interesting finding reveals that either in assessment or prediction, merely leveraging the modality of inflammation, liver function, or blood lipids data, etc., overwhelms any other single-modal input. It suggests that some test items in the inflammation modality and the liver function modality, such as C-reactive protein (CRP), hypersensitive C-reactive protein (hsCRP), γ-glutamyltransferase (GGT), and Albumin (ALB), are potential biomarkers in distinguishing COVID-19 infections.

## 8. Conclusion

In this paper, we have conceived and implemented a multistage, multimodal deep learning (MMDL) model to assess the disease severity and forecast the disease progression of patients with COVID-19. In summary, the novelty of MMDL embodies sequential stage-wise learning with multimodal inputs. MMDL shows the advantage of studying whole courses of the disease compared to single-stage learning. Also, mining the multimodal clinical data can provide significant performance gains over using single-modal data only. Some potential biomarkers have been identified in the control experiment, such as C-reactive protein (CRP) and hypersensitive C-reactive protein (hsCRP) of the inflammation modality, and γ-glutamyltransferase (GGT) and Albumin (ALB) of the liver function modality. A strong correlation is seen between these potential biomarkers and the assessment/prediction results. In addition, we have deployed the prototype of the MMDL model in Chongqing Public Health Center, China, to test MMDL's robustness to the new variants of COVID-19 and collect more clinical data for further incremental training.

## Data availability statement

The datasets presented in this article are not readily available because according to China's COVID-19 regulation and policy, any data related to patients with COVID-19 is not allowed to be disclosed publicly. Requests to access the datasets should be directed to cliff.zhuo.li@gmail.com.

## Author contributions

ZL conceived of the presented idea, designed the model, performed the implementation wrote the manuscript, and also encouraged. RX and YS to conduct the data preprocessing work, supervised the findings of this work and were mainly responsible for data cleaning, preprocessing, experiment setup, doing extensive comparison experiments, and analyzed the obtained results. JC contributed to the idea of overall model design and proofreading of the final version of the manuscript. BW provided insights on analyzing the clinical data and finding potential hall markers of infection of COVID-19 from the perspective of a physician. YZ and SL helped collect the multistage multimodal clinical data of examination and lab test results, facilitated the deployment of the prototype of the prediction model, and handled other routine work. All authors discussed the results and contributed to the final manuscript.

## Funding

This research is supported by the Ph.D., Scientific Research Sharing Foundation of the Chongqing University of Posts and Telecommunications (Project No. E012A2022026) and the Chongqing Key Special Program for Technology Innovation and Application Development (Project No. cstc2020jscx-fyzxX0023).

## Conflict of interest

The authors declare that the research was conducted in the absence of any commercial or financial relationships that could be construed as a potential conflict of interest.

## Publisher's note

All claims expressed in this article are solely those of the authors and do not necessarily represent those of their affiliated organizations, or those of the publisher, the editors and the reviewers. Any product that may be evaluated in this article, or claim that may be made by its manufacturer, is not guaranteed or endorsed by the publisher.
